# Genome sequences of BCG Pasteur ATCC 35734 and its derivative, the vaccine candidate BCGΔBCG1419c

**DOI:** 10.1186/s12864-023-09169-9

**Published:** 2023-02-10

**Authors:** Giuseppe D’Auria, Yordan Hodzhev, Michel de Jesús Aceves-Sánchez, Andrés Moya, Stefan Panaiotov, Mario Alberto Flores-Valdez

**Affiliations:** 1 Sequencing and Bioinformatics Service, Foundation for the Promotion of Health and Biomedical Research of Valencia Region, FISABIO, Valencia, Spain; 2 National Center of Infectious and Parasitic Diseases, 1504 Sofia, Bulgaria; 3 Biotecnología Médica y Farmacéutica, Centro de Investigación y Asistencia en Tecnología y diseño del Estado de Jalisco, A.C., Av. Normalistas 800, Col. Colinas de la Normal, 44270 Guadalajara, Jalisco Mexico; 4 Area of Genomics and Health, Foundation for the Promotion of Sanitary and Biomedical Research of Valencia Region (FISABIO-Public Health), Valencia, Spain; 5 CIBER in Epidemiology and Public Health (CIBERESP), Instituto de Salud Carlos III, Madrid, Spain; 6 Instituto de Biologia Integrativa de Sistemas, Universitat de València y Consejo Superior de Investigaciones Científicas, Valencia, Spain

**Keywords:** BCG, Vaccine, Pasteur, Genomic analysis, BCGΔBCG1419c

## Abstract

**Background:**

Bacillus Calmette–Guérin (BCG) remains the only vaccine to prevent tuberculosis (TB) during childhood, with relatively low to no efficacy against pulmonary TB in adolescents and adults. BCG consists of close to 15 different substrains, where genetic variations among them might contribute to the variable protective efficacy afforded against pulmonary TB. We have shown that the vaccine candidate, BCGΔBCG1419c, which is based on BCG Pasteur, improved protection against chronic TB in murine models, as well as against pulmonary and extrapulmonary TB in guinea pigs. Here, to confirm deletion of the *BCG1419c* gene and to detect possible genetic variations occurring as a consequence of the spontaneous mutations that may arise during in vitro culture of mycobacteria, the genomes of BCG Pasteur ATCC 35734 and its isogenic derivative, BCGΔBCG1419c, were sequenced and subjected to a comparative analysis between them and against BCG Pasteur 1173P2.

**Results:**

The complete catalog of variants in genes relative to the reference genome BCG Pasteur 1173P2 (GenBank NC008769) showed that the parental strain BCG Pasteur ATCC 35734, from which the mutant BCGΔBCG1419c originated, showed five synonymous mutations, three missense mutations, and five codon insertions, whereas the BCGΔBCG1419c mutant reported the same changes. When BCG Pasteur ATCC 35734 and BCGΔBCG1419c were compared, we confirmed that the latter was devoid of the *BCG1419c* gene, with only one unanticipated SNP at position 2, 828, 791  which we consider has no role in vaccine properties reported thus far.

**Conclusion:**

We provide evidence that the mutagenesis performed to remove *BCG1419c* from BCG Pasteur ATCC 35734 solely deleted this gene, and that compared with the reference strain BCG Pasteur 1173P2, few changes were present confirming that they are BCG Pasteur strains, and that changes in immunogenicity or efficacy observed thus far in BCGΔBCG1419c are most likely derived solely from the elimination of the *BCG1419c* gene.

## Background

Bacille Calmette-Guérin (BCG) is an attenuated strain of *Mycobacterium bovis* and is the only available vaccine against tuberculosis (TB). Since its introduction 100 years ago, it is estimated that more than 3 billion individuals have received BCG and over 100 million doses of BCG are administered annually to reduce TB burden worldwide. BCG is generally safe and can protect children against disseminated disease, including meningitis; in fact, a very recent meta-analysis suggests that BCG vaccination at birth is effective at preventing TB in young children, but as previously thought, is ineffective in adolescents and adults [1].

BCG typically refers to several substrains, each having genomic differences concerning reference strains [2]. Close to 50 production substrains have been used at one time or another in various parts of the world [3], including the major BCG vaccines in current use (BCG-Danish, -Glaxo, -Russia, and -Japan), which have recently been shown to differ in their viability, RNA content and capacity to induce ex vivo immune responses [4].

Considering that the relative protective efficacy of BCG substrains is a matter of debate [5], coupled with the inefficacy of BCG to protect adolescents and adults against pulmonary TB, there is an urgent need for novel and improved vaccines that could replace or boost the protective effect produced upon immunization with BCG.

In this regard, we developed the BCGΔBCG1419c vaccine candidate based on BCG substrain Pasteur. The second-generation version of BCGΔBCG1419c, devoid of antibiotic markers and based on Pasteur ATCC 35734 was recently shown to improve protection of C57BL/6 mice against the Haarlem strain *M. tuberculosis* M2 in reducing lung pathology compared with BCG Pasteur ATCC 35734 [6]. Also, BCGΔBCG1419c improved protection in guinea pigs against pulmonary and extrapulmonary TB better than parental BCG [7], and it showed variations in its cellular and secreted proteome compared with parental BCG [8].

Here, to identify potential genomic polymorphisms in BCGΔBCG1419c compared with its parental BCG Pasteur ATCC 35734 substrain and the reference genome of BCG Pasteur 1173P2, as well as to evaluate whether additional genetic events (insertion/deletion) other than the targeted deletion of the *BCG1419c* gene, we have obtained the whole genome sequences (WGS) of the BCG ATCC 35734 and that of the BCGΔBCG1419c strains. Obtained results were assembled and compared with the genome of the reference strain BCG Pasteur 1173P2 to identify eventual major genomic rearrangements. A mapping strategy was also used to evaluate SNPs/InDels variability acquired.

Thus, we confirmed that BCGΔBCG1419c has a single deletion of the *BCG1419c gene* and identified novel genomic polymorphisms of both BCG Pasteur ATCC 35734 and BCGΔBCG1419c compared with BCG Pasteur 1173P2.

## Results

A total of 7,772,967 and 7,577,201 paired ends raw reads have been obtained from the BCG Pasteur ATCC 35734 and BCGΔBCG1419c strains, respectively. The two genome reads datasets have been mapped against the reference genome *M. tuberculosis* BCG str. Pasteur 1173P2, obtaining an average coverage of 388x and 379x, respectively.

**Fig. 1:**
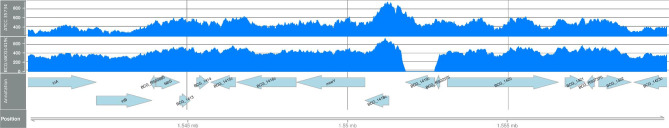
Schematic representarion showing the genomic region upstream and downstream the deletion of the BCG1419c gene. The upper panels shows the region present in wild type BCG Pasteur ATCC 35734, the middle panel, the region present in BCG?BCG1419c, and the bottom panel shows gene names when they have an annotation available.

The parental strain BCG Pasteur ATCC 35734, from which the mutant BCGΔBCG1419c originated, belonged to the BCG str. Pasteur substrain . The sequenced BCG Pasteur ATCC 35734 strain showed five synonymous mutations, three missense mutations, and four codon insertions compared with the BCG str. Pasteur 1173P2 (Table 1). The BCGΔBCG1419c mutant, on the other hand, had the same mutations as BCG Pasteur ATCC 35734 (Table 1) plus the deletion of *BCG1419*c, which is annotated as cyclic diguanylate phosphodiesterase. Further to this, we found an unanticipated SNP at position 2, 828, 791. In Table 1, the column “Evidence” indicates the frequencies (sequences) of the nucleotides in the reference (REF) genome with respect to its alternative (ALT, mutation) genome. Figure 1 shows the region surrounding the deletion.

Specifically, synonymous changes were found for PE_PGRS7, PE_PGRS28, and PE_PGRS53, whereas changes possibly affecting function were found for PE-PGRS family protein Wag22b, PE_PGRS43b, PE_PGRS53, and PE_PGRS57 (Table 1). Regarding non-PE_PGRS family genes, we found a synonymous change in *BCG_2507c*, which encodes for a LuxR-family transcriptional regulator, and we found two disruptive in-frame insertions, one in *BCG_3499c* and another in *BCG_3517* (Table 1).

## Discussion

Previously, spontaneous heterogeneity of BCG seed lots and commercial vaccines used during vaccine production was demonstrated in the BCG Tokyo-172 vaccine strain as determined by deep-sequencing [9]. Because of this reason that may impact on immunogenicity and/or efficacy of protection of TB vaccines in general, we decided to determine the WGS of our BCGΔBCG1419c vaccine candidate and its parental strain, BCG Pasteur ATCC 35734, a passage “zero” strain as obtained from ATCC. BCG Pasteur 35734 was passaged 3 times in our lab, and BCGΔBCG1419c was passaged 9  times by the time genomic DNA was obtained from them for WGS. We cannot rule out the fact that spontaneous mutations could arise during subsequent passages of our BCGΔBCG1419c vaccine candidate, which could lead to changes affecting efficacy of protection, as we have hypothesized to occur for other vaccine strains where the global regulator gene *phoP* is affected [10]. With the current availability of WGS, it would be convenient to monitor these possible changes over time to make sure that the vaccine strain maintains or not its reported properties.

In our study, most changes detected in both BCG Pasteur ATCC 35734 and its isogenic derivative BCGΔBCG1419c compared with BCG Pasteur 1173P2 were found in PE family genes , including *PE_PGRS7*, *PE_PGRS28*, PE-PGRS family *Wag22b*, *PE_PGRS43b*, *PE_PGRS53*, and *PE_PGRS57*. From these, *PE_PGRS43*, *PE_PGRS53*, and *PE_PGRS57* have been found in infected guinea pig lungs, and overall, this family has been suggested to play important roles in virulence [11].

*Rv2488c* (*mclx3*), homologous to *BCG_2507c*, showed a synonymous variant among our strains and BCG Pasteur 1173P2 (Table 1). *Rv2488c* presented a higher tendency for pseudogenization among isolates from patients born on the Western Pacific area, and from isolates causing extra-pulmonary infections [12]. *Rv3433c*, homologous to *BCG_3499c*, presented a disruptive in-frame insertion among our strains and BCG Pasteur 1173P2 (Table 1). Rv3433c was identified by mass spectrometry in *M. tuberculosis* H37Rv-infected guinea pig lungs at 90 days but not 30 days [13].

**Table 1:** Summary of changes detected in the genomes of BCG Pasteur ATCC 35734 and its isogenic derivative BCGΔBCG1419c compared with BCG Pasteur 1173P2

**BCG Pasteur ATCC 35734**														
CHROM	POSITION	TYPE	REF	ALT	EVIDENCE	FTYPE	STRAND	NT_POS	AA_POS	EFFECT	LOCUS_TAG	BCG 1172P2 GENE	H37Rv GENE	PRODUCT
NC_008769	705623	complex	GTGG	ATGC	ATGC:125 GTGG:0	CDS	-	1176/3912	391/1303	synonymous_variant c.1173_1176delCCACinsGCAT p.393	BCG_RS03180	*BCG_0623c*	PE_PGRS7	PE family protein
NC_008769	1344671	mnp	CG	GC	GC:119 CG:0									
NC_008769	1661323	snp	G	C	C:12 G:0	CDS	-	1926/2223	642/740	synonymous_variant c.1926C > G p.Gly642Gly	BCG_RS07850	*BCG_1513c*	PE_PGRS28	PE family protein
NC_008769	2002385	complex	G	CGGC	CGGC:61 G:3	CDS	-	666/2690	222/895	disruptive_inframe_insertion&synonymous_variant c.666delCinsGCCG p.Thr222_Val223insPro	BCG_RS09290	*BCG_1799c*	PE-PGRS family protein wag22b	PE family protein
NC_008769	2764157	snp	T	A	A:260 T:0	CDS	-	459/3342	153/1113	synonymous_variant c.459A > T p.Thr153Thr	BCG_RS12955	*BCG_2507c*	Rv2488c	LuxR family transcriptional regulator
NC_008769	2766103	complex	G	CGGC	CGGC:103 G:0	CDS	-	3930/4972	1310/1656	missense_variant&disruptive_inframe_insertion c.3930delCinsGCCG p.Cys1310delinsTrpPro	BCG_RS12965	*BCG_2509c*	PE_PGRS43b	PE family protein
NC_008769	3833488	ins	G	GGCC	GGCC:141 G:9	CDS	-	1298/1419	433/472	disruptive_inframe_insertion c.1298_1299insGGC p.Ala433dup	BCG_RS17970	*BCG_3499c*	Rv3433c	Bifunctional ADP-dependent NAD(P)H-hydrate dehydratase/NAD(P)H-hydrate epimerase
NC_008769	3854969	ins	G	GGTC	GGTC:289 G:0	CDS	+	783/786	261/261	disruptive_inframe_insertion c.782_783insTCG p.Gly261_Ter262insArg	BCG_RS18060	*BCG_3517*	Cut3	Cutinase family protein
NC_008769	3907860	snp	A	G	G:81 A:0	CDS	+	1795/4119	599/1372	missense_variant c.1795A > G p.Asn599Asp	BCG_RS18320	*BCG_3571*	PE_PGRS53	PE family protein
NC_008769	3908180	snp	C	T	T:70 C:0	CDS	+	2115/4119	705/1372	synonymous_variant c.2115C > T p.Gly705Gly	BCG_RS18320	*BCG_3571*	PE_PGRS53	PE family protein
NC_008769	3927720	snp	A	G	G:13 A:0	CDS	+	1792/3228	598/1075	missense_variant c.1792A > G p.Thr598Ala	BCG_RS18350	*BCG_3577*	PE_PGRS57	PE family protein
**BCGΔBCG1419c**														
CHROM	POSITION	TYPE	REF	ALT	EVIDENCE	FTYPE	STRAND	NT_POS	AA_POS	EFFECT	LOCUS_TAG	BCG 1172P2 GENE	H37Rv GENE	PRODUCT
NC_008769	705623	complex	GTGG	ATGC	ATGC:137 GTGG:0	CDS	-	1176/3912	391/1303	synonymous_variant c.1173_1176delCCACinsGCAT p.393	BCG_RS03180	*BCG_0623c*	PE_PGRS7	PE family protein
NC_008769	1344671	mnp	CG	GC	GC:91 CG:0									
NC_008769	1661323	snp	G	C	C:16 G:0	CDS	-	1926/2223	642/740	synonymous_variant c.1926C > G p.Gly642Gly	BCG_RS07850	*BCG_1513c*	PE_PGRS28	PE family protein
NC_008769	2002385	complex	G	CGGC	CGGC:61 G:0	CDS	-	666/2690	222/895	disruptive_inframe_insertion&synonymous_variant c.666delCinsGCCG p.Thr222_Val223insPro	BCG_RS09290	*BCG_1799c*	PE-PGRS family protein wag22b	PE family protein
NC_008769	2764157	snp	T	A	A:266 T:0	CDS	-	459/3342	153/1113	synonymous_variant c.459A > T p.Thr153Thr	BCG_RS12955	*BCG_2507c*	Rv2488c	LuxR family transcriptional regulator
NC_008769	2766103	complex	G	CGGC	CGGC:110 G:0	CDS	-	3930/4972	1310/1656	missense_variant&disruptive_inframe_insertion c.3930delCinsGCCG p.Cys1310delinsTrpPro	BCG_RS12965	*BCG_2509c*	PE_PGRS43b	PE family protein
NC_008769	2828791	snp	G	A	A:155 G:0									
NC_008769	3833488	ins	G	GGCC	GGCC:159 G:6	CDS	-	1298/1419	433/472	disruptive_inframe_insertion c.1298_1299insGGC p.Ala433dup	BCG_RS17970	*BCG_3499c*	Rv3433c	Bifunctional ADP-dependent NAD(P)H-hydrate dehydratase/NAD(P)H-hydrate epimerase
NC_008769	3854969	ins	G	GGTC	GGTC:279 G:0	CDS	+	783/786	261/261	disruptive_inframe_insertion c.782_783insTCG p.Gly261_Ter262insArg	BCG_RS18060	*BCG_3517*	Cut3	Cutinase family protein
NC_008769	3907860	snp	A	G	G:37 A:0	CDS	+	1795/4119	599/1372	missense_variant c.1795A > G p.Asn599Asp	BCG_RS18320	*BCG_3571*	PE_PGRS53	PE family protein
NC_008769	3908180	snp	C	T	T:99 C:1	CDS	+	2115/4119	705/1372	synonymous_variant c.2115C > T p.Gly705Gly	BCG_RS18320	*BCG_3571*	PE_PGRS53	PE family protein
NC_008769	3927720	snp	A	G	G:11 A:0	CDS	+	1792/3228	598/1075	missense_variant c.1792A > G p.Thr598Ala	BCG_RS18350	*BCG_3577*	PE_PGRS57	PE family protein

The column “Evidence” indicates the frequencies (sequences) of the nucleotides in the reference (REF) with respect to its alternative (ALT, mutation) genome

As for *BCG_3517*, this gene also showed a disruptive in-frame insertion among our strains and BCG Pasteur 1173P2 (Table 1). Transcripts from its homologous gene *cut3* (*Rv3451*) were increased in a *mce1* mutant, along with transcripts of *mmpL3*, *fas*, *kasA*, *kasB* and *acpM*, involved in mycolic acids transport and metabolism [14].

Overall, considering that other than deletion of *BCG1419c*, BCGΔBCG1419c differs from BCG Pasteur ATCC 35734 only in a SNP at position 2,828,791, this support the notion that the improved efficacy and changes to the proteome we have reported for the BCG ATCC 35734-derived version of BCGΔBCG1419c [6–8] are most likely the sole consequence of the gene deletion we created. The SNP at position 2,828,791 is located in the intergenic region of what would be homologous to *BCG_2563* (annotated as hypothetical alanine rich protein) and *BCG_2564* (a conserved hypothetical protein with an α/β hydrolase 8 family protein). Considering its location and the predicted functions of these genes, we hypothesize that this SNP plays no role in vaccine efficacy reporte thus far. 

## Conclusion

Recently, comparative studies of genomic variations in BCG strains at their different stages of production and utilization (production strains, their seeds, administered vaccine lots) was suggested to potentially provide data to better understand the bases of vaccine efficacy and adverse reactions of present and future BCG-based vaccines [15]. Here, we provide the WGS of our vaccine candidate, BCGΔBG1419c and its parental strain, BCG Pasteur ATCC 35734. Our analysis show that BCGΔBCG1419c differs from BCG Pasteur ATCC 35734 only in a SNP at position 2,828,791, hereby supporting the notion that the improved efficacy we have observed for BCGΔBG1419c in preclinical models are most likely the sole consequence of the gene deletion of* BCG1419c* we created and support further development of this vaccine candidate.

## Methods

### Construction of the BCGΔBCG1419c mutant

BCG Pasteur ATCC 35734 was used as parental strain to promote homologous recombination to create the antibiotic-less version of BCGΔBCG1419c as already described in detail [8]. Succinctly, sequences upstream and downstream of *BCG1419c* were amplified by PCR and cloned into pUCHyg (a kind gift from Dr. Yi-.

Cheng Sun), sequences were verified, and this plasmid was transformed by electroporation into BCG Pasteur ATCC 35734 harboring pJV53 (a kind gift from Dr. Graham Hatfull). Recombination and successful mutagenesis was verified as described [16].

### Genomic DNA extraction

BCG Pasteur ATCC 35734 and its isogenic derivative, BCGΔBG1419c, were cultured in Middlebrook 7H9 broth, supplemented with 10% OADC, at 37ºC, 100 rpm, until OD600nm 0.8. Then, cell pellets were obtained by centrifugation at 3,200 x g for 10 min. The bacterial pellets were resuspended in SET buffer (0.25 M sucrose, 0.05 M EDTA, 0.03 M Tris) and lysozyme (50 mg/mL) was added followed by incubation overnight at 37ºC. RNAse A was added (10 mg/mL) and incubated at 37ºC for 30 min, followed by the addition of proteinase K (1 mg/mL) to incubate at 55ºC for 2 h. A phenol-chloroform–isoamyl alcohol extraction step was performed, to separate the aqueous phase and add 0.1 V of 3 M sodium acetate (pH 5.2), and 0.7 volume of isopropanol for precipitation performed by centrifugation at 16,000 x g, 4ºC for 30 min. The DNA pellets were washed with 7% ethanol, the supernatant discarded, and the pellets were air-dried to resuspend in molecular biology-grade water finally. This protocol was adapted from that described by van Soolingen et al. [17].

### Library Preparation and sequencing

The genomic DNA was randomly sheared into short fragments using enzymes provided in the Nextera XT DNA Library Preparation Kit (Illumina, USA) following the manufacturer’s protocol to achieve equimolar pools of each library sample. The obtained fragments were end-repaired, A-tailed, and further ligated to Illumina adapters by “tagmentation”. The fragments with adapters were PCR amplified, size selected using MPure XP Beads (Beckman Coulter, USA), and purified. The size distribution of fragments was checked using an Agilent DNA High Sensitivity chip (2100 Bionanalyzer, Agilent, USA). An Illumina library was prepared with the Nextera DNA Flex kit and Nextera DNA CD indexes, and 2 × 150-bp sequencing was performed on a MiSeq sequencer using the MiSeq reagent kit v.3 (Illumina, USA).

### Sequencing quality assessment

Illumina output files in FASTQ format were loaded into Geneious Prime software (v.2021.2.2) and trimmed with the BBDuk plugin (v.1.0, https://sourceforge.net/projects/bbmap/). Adapters on the right and low-quality ends (quality below 20%) were trimmed, while reads shorter than 200 bp were discarded. Then, the reads were subjected to preprocessing (https://www.geneious.com/tutorials/map-to-reference/). The genomes were assembled by using the “Map to reference” tool. BCG Pasteur 1173P2 (GenBank accession no. NC008769) was used as a reference genome. A consensus sequence from aligned reads was extracted. We visually confirmed the circular genomes of the *M. bovis* BCG strains by assessing the reads spanning the junction between the two linearized ends and overlapping with them. Annotation was generated with the NCBI Prokaryotic Genome Annotation Pipeline (PGAP) (v.4.13) [18–20].

The sequencing data belonging to the BCG Pasteur ATCC 35734 (NCBI Locus tag: CP109681; https://www.ncbi.nlm.nih.gov/nuccore/CP109681) strain and its isogenic derivative, BCGΔBCG1419c (NCBI Locus tag: CP110223; https://www.ncbi.nlm.nih.gov/nuccore/CP110223), were mapped against the reference genome *Mycobacterium* bovis BCG Pasteur 1173P2 (NCBI Locus tag: NC_008769; https://www.ncbi.nlm.nih.gov/nuccore/NC_008769). The mapping process was carried out using the Snippy pipeline (V 4.6.0; Seemann 2015, https://github.com/tseemann/snippy). Genomes alignment was visualized using Mauve program [21] (snapshot 2015-02-13).

## Data Availability

The raw sequence reads can be found as CP110223.1 - *Mycobacterium tuberculosis* variant bovis strain BCG delta BCG1419c mutant chromosome. https://www.ncbi.nlm.nih.gov/nuccore/CP110223, and CP109681.1 - *Mycobacterium tuberculosis* variant bovis strain BCG Pasteur ATCC 35,734 chromosome https://www.ncbi.nlm.nih.gov/nuccore/CP109681. All data generated or analyzed during this study are included in this article and its supplementary information files.

## Abbreviations

BCG: Bacille Calmette-Guérin
TB: Tuberculosis
SNP: Single nucleotide polymorphisms
WGS: Whole-genome sequencing
CDS: Coding DNA sequences
